# Quantitative computed tomography phenotypes, spirometric parameters, and episodes of exacerbation in heavy smokers: An analysis from South America

**DOI:** 10.1371/journal.pone.0205273

**Published:** 2018-10-11

**Authors:** Marcelo Cardoso Barros, Bruno Hochhegger, Stephan Altmayer, Guilherme Watte, Matheus Zanon, Ana Paula Sartori, Daniela Cavalet Blanco, Gabriel Sartori Pacini, Jose Miguel Chatkin

**Affiliations:** 1 Department of Pulmonology, Pontíficia Universidade Católica do Rio Grande do Sul, Porto Alegre, RS, Brazil; 2 Department of Radiology, Irmandade Santa Casa de Misericórdia de Porto Alegre, Porto Alegre, RS, Brazil; University of Calcutta, INDIA

## Abstract

**Objective:**

To evaluate the quantitative computed tomography (QCT) phenotypes, airflow limitations, and exacerbation-like episodes in heavy smokers in Southern Brazil.

**Methods:**

We enrolled 172 smokers with a smoking history ≥30 pack-years who underwent pulmonary function tests (PFTs) and CT scan for lung cancer screening. Patients were classified regarding airflow limitation (FEV_1_/FVC <0.7 forced expiratory volume in 1 second/forced vital capacity) and the presence of emphysema on the QCT. The QCT were analyzed in specialized software and patients were classified in two disease-predominant phenotypes: emphysema-predominant (EP) and non-emphysema-predominant (NEP). EP was determined as ≥6% of percent low-attenuation areas (LAA%) with less than -950 Hounsfield units. NEP was defined as having a total LAA% of less than 6%.

**Results:**

Most of our patients were classified in the EP phenotype. The EP group had significantly worse predicted FEV_1_ (60.6 ±22.9 vs. 89.7 ±15.9, p <0.001), higher rates of airflow limitation (85.7% vs. 15%; p <0.001), and had more exacerbation-like episodes (25.8% vs. 8.3%, p <0.001) compared to the NEP group. Smoking history, ethnicity, and BMI did not differ between the groups. The total LAA% was the QCT parameter with the strongest correlation to FEV_1_ (r = -0.669) and FEV_1_/FVC (r = -0.787).

**Conclusions:**

Heavy smokers with the EP phenotype on QCT were more likely to have airflow limitation, worse predicted FEV_1,_ and a higher rate of exacerbation-like episodes than those with the NEP phenotype. Approximately 23% of patients with no airflow limitation on PFTs were classified in EP phenotype.

## Introduction

Chronic obstructive pulmonary disease (COPD) is a complex syndrome characterized by persistent and progressive airflow obstruction with several pulmonary and extrapulmonary components [1–3]. It is defined by guidelines from the global initiative for chronic obstructive lung disease (GOLD) using both clinical and pulmonary function criteria [1]. The pulmonary function criterion is persistent airflow limitation by spirometry, which is defined as a ratio of forced expiratory volume in 1 second (FEV_1_) over the forced vital capacity (FVC) less than 0.7. Although spirometry is easily available at a low cost, the FEV_1_/FVC criterion might not adequately detect all heterogeneity in this syndrome [2], such as its morphological components (emphysema and airway remodeling) [3], and incipient or subclinical forms [4]. As a consequence, many individuals might not meet the threshold necessary for a COPD diagnosis, despite the presence of airway disease and a higher risk for COPD exacerbation-like episodes [5]. This subset of patients with normal spirometry, but already established airway disease, could benefit from medical care if identified earlier.

Quantitative computed tomography (QCT) allows objective and noninvasive identification and quantification of emphysema earlier than spirometry, and possibly before the emergence of symptoms [6]. Assessment of airway morphology, lung parenchyma and other underlying conditions can be used to define the patient’s predominant phenotype: emphysema-predominant (EP), and non-emphysema predominant (NEP) [7, 8]. The categorization of COPD into these structural and functional phenotypes plays an important role in determining outcomes related to exacerbations, antimicrobial therapy, decline in pulmonary function, and mortality [5,8]. Thus, early diagnosis is important to avoid the progression of irreversible lesions in the parenchyma, with several studies showing an association of increased emphysema percentage on tomography with loss of physical capacity and increased mortality [9,10].

Considering that COPD is a heterogeneous condition, the characterization of phenotypic tomographic in heavy smokers has been the focus of previous comprehensive studies especially in North America, Europe and Asia [11–16]. Except for the PLATINO study [17], characterization of COPD in the Latin America population are still underrepresented in the literature–especially regarding QCT phenotypes. Thus, the goal of this study was to evaluate CT phenotypes, pulmonary function, and respiratory outcomes in heavy smokers in Southern Brazil.

## Materials and methods

### Study population

**Fig 1:**
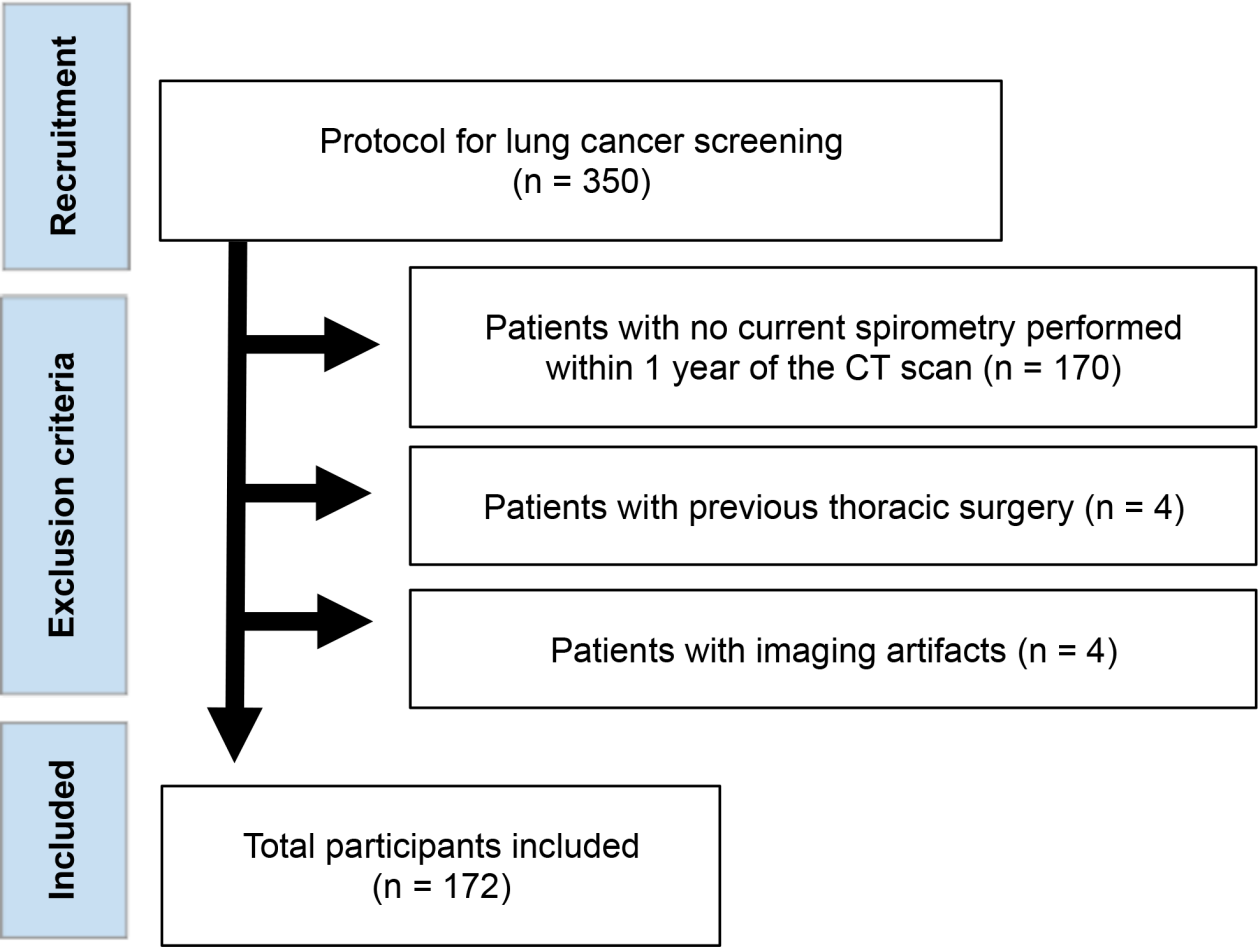
Flow diagram showing the inclusion criteria of the study population.

We retrospectively identified 350 who were undergoing screening for lung cancer at a tertiary hospital in Southern Brazil between January 2014 and January 2016. Patients were included if they were ≥50 years, smoking history of at least 30 pack-years, and had both spirometry and chest CT performed within 1 year. Both active and former smokers (who quit smoking within 10 years) were included. Exclusion criteria were the presence of respiratory motion artifacts in CT images, a history of thoracic surgery, or severe heart disease (Fig 1). Baseline characteristics (age, sex, smoking history, ethnicity, and body mass index) and the occurrence of COPD exacerbation-like episodes in the preceding 12 months were collected for all patients. These episodes were defined using the GOLD criteria for COPD exacerbation as an acute worsening of respiratory symptoms that resulted in additional therapy [18, 19]. Patients were classified as smokers with or without airflow limitations (FEV_1_/FVC ratio < 0.7 or FEV_1_/FVC ≥ 0.7, respectively) according to the current GOLD pulmonary function criteria [1]. This retrospective study was approved by the institutional review board of the Pontificia Universidade Catolica do Rio Grande do Sul with waiver of consent.

### Computed tomography assessment

Chest CTs were performed using a 16-slice multidetector scanner (GE BrightSpeed; GE Healthcare, Waukesha, WI, USA) without intravenous contrast. Images were obtained as a single acquisition during inspiration using the following parameters: power of 120 KVp, current of 60 mA, gantry rotation time of 0.5 s, pitch of 1.375, collimation of 20 mm, increments of 5 mm, and 1.25-mm-thick reconstructions. The effective radiation doses ranged from 0.8 to 1.3 mSv, and the dose-length product was 69 to 86 mGy·cm. The protocol of the QCT was followed according to the recommendations by Newell et al [20].

### Computed tomography quantification

Quantitative analysis of CT images was performed using Airway Inspector software (http://www.airwayinspector.org/) by two thoracic radiologists experienced with the software. The analysis included data for airway wall thickness (AWT) and the percentage of airway wall area (WA%) obtained from the third to fifth-generation bronchi in the upper and lower lobes. One bronchus per pulmonary lobe was randomly selected and it was necessary to manually draw the bronchial wall for the software to calculate the airway area and the airway wall thickness.

We established two disease-predominant phenotype groups based on the areas of percentage of low-attenuation areas (LAA%), determined as those with less than -950 HU in the lung parenchyma. The EP group was defined as patients with ≥6% of LAA in the total lung parenchyma, while the NEP group were those patients with <6% of LAA [8]. The cutoff set at -950 HU has demonstrated a strong macroscopic and microscopic correlation between clinical emphysema and imaging findings in several studies [21,22]. The LAA% was automatically calculated by the specialized software.

### Pulmonary function tests

Spirometry was performed using a Quark pulmonary function test (PFT) system (Cosmed, Rome, Italy) in accordance with guidelines of the European Respiratory Society (ERS) and American Thoracic Society (ATS) [23]. All predictive equations were performed and validated in Brazilian cohorts [24,25]. The airflow limitation criteria were defined according to the current GOLD guidelines: FEV_1_/FVC less than 0.7 measured after bronchodilator administration (inhalation of 400 μg of salbutamol). Patients were classified as smokers with or without airflow limitations (FEV_1_/FVC ratio < 0.7 or FEV_1_/FVC ≥ 0.7, respectively) according to the current GOLD pulmonary function criteria.

### Statistical analysis

The data are presented as absolute and relative frequencies (percentages) for categorical variables or the mean and standard deviation for numerical variables. We assessed the associations between variables using exact Fisher’s test or the chi-square test. The student *t* test or an unequal variance *t* test was used when comparing continuous variables. Spearman's rank correlation was used to assess linear associations. Statistical significance was accepted at p < 0.05. Correlations between variables were measured using Spearman’s correlation coefficient, and the following ranges were defined: 0.00 to 0.19, very weak; 0.20 to 0.39, weak; 0.40 to 0.59, moderate; 0.60 to 0.79, strong; and 0.80 to 1.00, very strong [26]. All statistical analyses were performed using the Statistical Package for the Social Sciences (PASW Statistics for Windows; Version 18.0. SPSS Inc., Chicago, IL, USA).

## Results

### Population characteristics

Our final sample was composed of 172 heavy smokers, and their baseline characteristics are presented in Table 1. In comparison to those with airflow limitations, the patients with no limitation were predominantly female (58.2% vs. 31.5%, p <0.001), were younger, had a lower smoking history (60.53 ±24.50 vs. 77.20 ±38.03, p = 0.009), and were less likely to have exacerbation-like episodes (9% patients vs. 26.6%, p = 0.004). Regarding the QCT, those with no airflow limitation had a lower degree of lung areas with low-attenuation (4.52 ±3.55% vs. 17.01 ±9.96%, p <0.001), lower lung volume (5.07 ±1.18 vs. 6.22 ±1.32 in liters, p<0.001), and percentage of wall area (63.42 ±6.09% vs. 66.94 ±5.53%, p <0.001). Most of the patients in the airflow limitation group were GOLD class 2 (66.6%).

**Table 1:** Demographic and quantitative CT characteristics stratified by airflow limitation.

	Total (n = 172)	FEV_1_/FVC <0.7 (n = 105)	FEV_1_/FVC ≥0.7 (n = 67)	p-value
Male (%)	100 (58)	72 (68.5)	28 (41.8)	0.001
Age (years)	63.3 ±6.0	64.6 ±6,10-	61.4 ±5.3	0.001
Body mass index (kg/m^2^)	26.96 ±5.69	26.32 ±5.43	27.95 ±5.97-	0.068
Smoking (pack-years)	70.70 ±34.32	77.20 ±38.03-	60.53 ±24.50	0.009
Exacerbation episodes (n)	34 (19.7)	28 (26.6)	6 (9)	0.004
QCT				
ULs LAA% -950 (%)	13.75 ±13.73	19.45 ±14.59	4.82 ±4.51	<0.001
Total LAA% -950 (%)	12.15 ±10.12	17.01 ±9.96-	4.52 ±3.55	<0.001
Total lung volume (L)	5.77 ±1.38	6.22 ±1.32-	5.07 ±1.18	<0.001
AWT	1.22 ±0.63	1.23 ±0.06-	1.21 ±0.06	0.140
WA%	65.56 ±5.99	66.94 ±5.53-	63.42 ±6.09	<0.001
GOLD classification				<0.001
No airflow limitation	67 (39)	0	67 (100)	
GOLD 1	0	0	0	
GOLD 2	70 (41)	70 (66.6)	0	
GOLD 3	33 (19)	33 (31.4)	0	
GOLD 4	2 (1)	2 (2)	0	

Data are presented as a “n” (%) or mean ± SD.

QCT, quantitative computed tomography; ULs, upper lobes; LAA%, percent of low-attenuation areas; AWT, airway wall thickness; WA%, wall area percent.

### CT phenotypes

Demographic, clinical, and QCT characteristics stratified according to the CT phenotype is shown in Table 2. The EP group comprised 112 patients, and the NEP group comprised 60. Those in the EP group were predominantly male, and on average older than NEP group (64.54 ± 6.04 years old vs. 61.25 ± 5.46 years old, p < 0.001). Smoking history, ethnicity, and body mass index were not different between groups. The EP group had significantly worse FEV_1_% (60.60 ±22.87 vs. 89.76±15.91, p < 0.001), and greater exacerbation-like episodes than the NEP (n = 29, 25.8% vs. n = 5, 8.3%; p = 0.005).

**Table 2:** Clinical and demographic characteristics of heavy smokers by QCT disease-dominant phenotype.

	Total (n = 172)	Disease-predominant phenotype		p-value
		EP (n = 112)	NEP (n = 60)	
Male, n (%)	100 (58)	77 (68.7)	23 (38.3)	<0.001
Age (years)	63.3±6.0	64.5±6.00	61.2±5.4	<0.001
Non-white	110 (64)	70 (62.5)	40 (66.6)	0.588
Body mass index (kg/m^2^)	26.96±5.690	26.79±5.94	27.29±5.210	0.548
Smoking (pack-years)	70.70±34.32	73.60±36.40	65.29±26.56	0.492
Exacerbation episodes (n)	34 (20)	29 (25.8)	5 (8.3)	0.005
Spirometry				
FEV_1_ (%predicted)	70.77±24.92	60.60±22.87	89.76±15.91	<0.001
FVC (%predicted)	90.53±22.27	87.07±24.20	97.01±16.44	0.002
FEV_1_/FVC (%)	63.82±16.54	55.62±13.30	79.10±9.780	<0.001
QCT				
ULs LAA% -950, (%)	13.75±13.73	19.49±13.89	3.04±1.99	<0.001
Total LAA% -950 (%)	12.15±10.12	17.03±9.36	3.04±1.64	<0.001
Total lung volume (L)	5.77±1.38	6.21±1.34	4.96±1.05	<0.001
AWT	1.22±0.63	1.22±0.06	1.23±0.06	0.454
WA%	65.56±5.990	65.95±5.430	64.81±6.920	0.278
GOLD classification				<0.001
No airflow limitation	67 (39)	16 (14.3)	51 (85.0)	
GOLD 1	0	0	0	
GOLD 2	70 (41)	61 (54.4)	9 (15.0)	
GOLD 3	33 (19)	33 (29.4)	0	
GOLD 4	2 (1)	2 (1.7)	0	

Data are presented as “n” (%) or mean ± SD.

FEV1, forced expiratory volume in the first second; FVC, forced vital capacity; QCT, quantitative computed tomography; ULs, upper lobes; LAA%, percent of low-attenuation areas; AWT, airway wall thickness; WA%, wall area percent; GOLD, global initiative for chronic obstructive lung disease.

**Fig 2:**
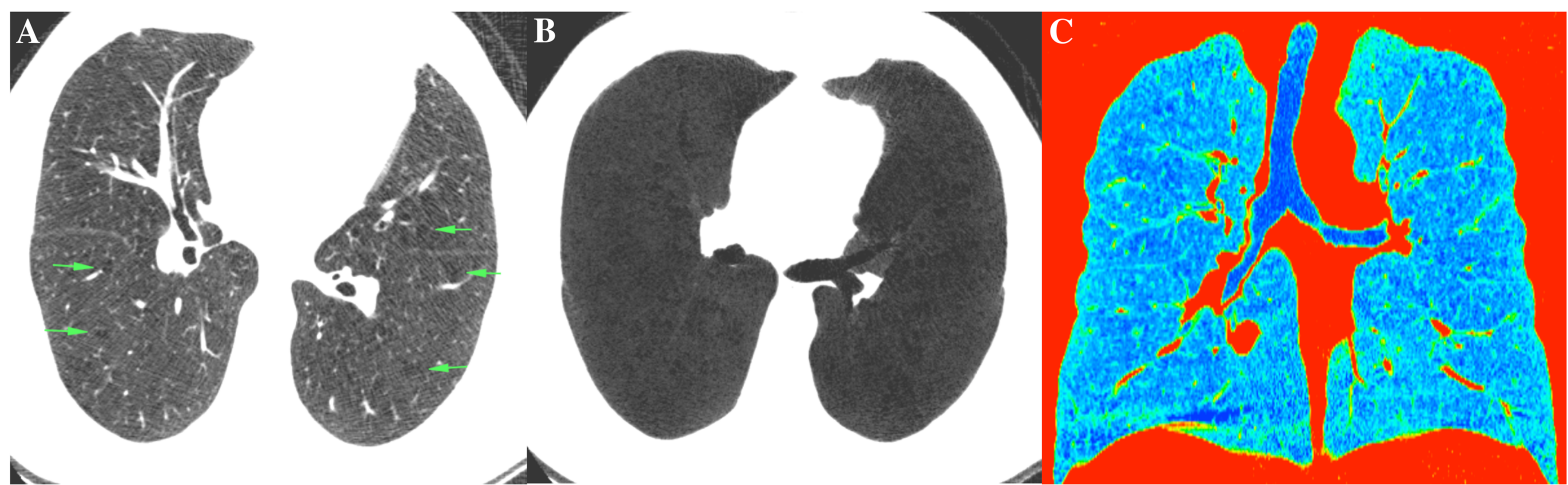
CT scan of a patient with FEV_1_/FVC = 73.4, but a total low-attenuation area of 18%. (A) Axial CT image demonstrating areas of emphysematous changes (arrowheads). (B) Axial MINIP reconstruction of the same patient shows a predominantly diffuse pattern of low-attenuation (C) Colored reconstruction of a coronal image demonstrating multiple low-attenuation areas in blue.

As predicted, the EP group had a greater percentage of LAA and total lung volume than the NEP, but the groups did not differ regarding AWT (p = 0.454) and WA% (p = 0.278). A total of 16 patients with no airflow limitation were classified as having the EP pattern, whereas 9 patients with airflow limitation were classified in the NEP group. In the EP group, the rate of exacerbation in patients with airflow limitation was 28.1% compared to 12.5% of those with no airflow limitation (p = 0.232). In the NEP group, the rates were 11.1% and 7.8%, respectively (p = 0.57). One of the patients with no airflow limitation on PFT but classified as having the EP phenotype is demonstrated in Fig 2.

### Correlation between pulmonary function and QCT parameters

The correlation coefficients of the PTFs and the QCT parameters are presented in Table 3. The total LAA% was the QCT variable with the strongest negative correlation with FEV_1_/FVC (r = -0.787, p < 0.01), and also the FEV_1_% (r = -0.669, p <0.01). The LAA% in the upper lobes also demonstrated a moderate to strong correlations. However, both AWT and WA% were only weakly correlated to the same variables.

**Table 3:** Correlations between lung function and QCT parameters (N = 172).

Variables	QCT			
	ULs LAA%	Total LAA%	AWT	WA%
Lung function test				
FEV_1_ (% of predicted)	-0.552**	-0.669**	-0.194**	-0.263**
FVC (% of predicted)	-0.208**	-0.322**	-0.120**	-0.156**
FEV_1_/FVC ratio (%)	-0.705**	-0.787**	-0.198**	-0.269**

Note. Spearman rank correlation

*P < 0.05 (2-tailed)

**P < 0.01 (2-tailed). Abbreviations: FEV1, forced expiratory volume in first second; FVC, forced vital capacity; ULs, upper lobes; LAA%, percent of low-attenuation areas; AWT, airway wall thickness; WA%, wall area percent; QCT, quantitative computed tomography.

## Discussion

The results indicate that heavy smokers have different QCT phenotypes that may or may not present with airflow limitation. The QCT phenotype of EP was associated with worse degrees of airway limitation, and also higher rate of exacerbation-like episodes. Also, we found that the QCT parameter that best correlates to pulmonary function was the total LAA%. A total of 23.8% patients that did not meet the criteria for airflow limitation were classified in the EP pattern, whereas 8.5% of patients with airflow limitation were classified as NEP. There was no difference in the smoking load between EP and NEP phenotypes.

Phenotyping heavy smokers according to QCT findings can be helpful in the early detection of COPD. Even symptomatic smokers often have normal pulmonary function tests, as alterations might appear only after major damage to the airways and lung parenchyma has occurred [5–8]. We showed that emphysematous changes could be detected in CT even in patients with no airway limitation. In our sample, 8.9% of the heavy smokers with no airflow limitation had exacerbation-like episodes, which is consistent with the literature [11,12].

The total LAA% was the QCT variable with the strongest negative correlations with FEV_1_/FVC ratio and FEV_1_ (r = -0.787 and -0.669, respectively). Similar correlation coefficients were also reported by Akira et al. [27]. In addition, patients with different proportions of LAA% were found to have similar values for FEV_1_, showing that equivalent spirometric findings can be associated with substantial, little, or no emphysema, as described by Lynch et al. [28]. In contrast, the FEV_1_/FVC ratio and FEV_1_ correlations were weak for AWT and WA%. Yahaba et al. [29] found significant correlations between FEV_1_ and airway measures in the QCT of the third- (segmental) to fifth-generation (sub-subsegmental) bronchi in patients without emphysema, but not for patients with emphysema. These results are likely explained by the loss of airway tethering in patients with emphysema, which may influence airway dimensions and therefore weakening the relationships with the degree of airflow impairment [29]. For this reason, the effects of emphysema should be considered when analyzing airway measurements in COPD. Most the patients in our sample displayed the EP phenotype, so this effect could have contributed to the weak correlations found between airway measurements in QCT and pulmonary function. Schroeder et al. [30] also found poor correlations between QCT airway parameters and FEV_1_ and FEV_1_/FVC values.

Subramanian et al. [7] found that the EP phenotype was associated with worse FEV_1_, including lung volume increases in QCT, which is consistent with our results. However, that author found greater WA% values in the airway-predominant group. We found no statistical differences between the EP and NEP groups regarding WA%, indicating that airway disease could also be present in the EP phenotype, which could suggest mixed disease. This mixed pattern could also explain the greater number of exacerbation-like episodes found in this group, which has been reported in another study [31]. On the other hand, the NEP phenotype presented the higher values of FEV_1_ and FEV_1_/FVC on spirometry, was predominantly composed of women, and had fewer exacerbation-like episodes.

In our study, we used the GOLD criteria to classify limitation of airflow. However, since FEV_1_ value decreases more than FVC with the age, using the lower limit of normal (LLN) can be a better alternative for patients, especially in elderly population [32]. Previous studies had already shown that the GOLD criteria may overdiagnose airflow limitation in COPD, while LLN is more prone to underdiagnose it [33]. In our service, the GOLD criteria are still standard of care to for early detection of airflow limitation, because we believe that it is important to institute therapy for the initial stages of the disease. Also, LLN identified fewer patients with relevant prognostic events, such as COPD exacerbation or mortality, than the GOLD criteria, reducing the number of elderly patients that could benefit from receiving a more intensive treatment to reduce those events [34].

Results from the COPDGene study found that the more advanced emphysema and greater AWTs were associated with the number of COPD exacerbations, independently of the degree of airflow obstruction represented by VEF_1_ [35]. Likewise, we observed a significantly greater number of exacerbation-like episodes in EP phenotype group, although we did not perform a multivariate analysis to test if this effect was independent of the presence of airway obstruction due to our small number of events. In the COPDGene cohort, patients with an LAA% ≥35% had an increasing likelihood of having episodes of exacerbation the higher were the areas of emphysema even after adjusting for airflow limitation. This could be important for the clinical management of smokers without COPD (airflow limitation) because episodes of exacerbation are associated with faster deterioration of pulmonary function parameters and increases in morbidity and mortality [36–38]. Smokers without airflow limitation are not included in the current COPD treatment guidelines because they do not fall within the definition of COPD. Thus, the first step in targeting disease progression in this subgroup is early detection, and CT can identify biomarkers for disease progression, as well as morphostructural and functional assessment [39].

Our study has some limitations. First, this is a retrospective study and therefore we could not include additional clinical parameters (medication list, adherence to treatment, comorbidities, smoking status on the follow-up) to be correlated with the CT phenotypes. Second, part of the initial sample could not be included in the study mostly due to lack of recent pulmonary function tests, which could be a source of bias. Third, we did not assess the carbon monoxide diffusion capacity or the expiratory CT images for our patients, as it is not routinely performed in lung cancer screening. Also, we did not account for the response to the bronchodilator challenge on spirometry, which could indicate asthma-COPD overlap in some patients. We did not evaluate the CT images qualitatively for the presence of associated interstitial lung diseases.

In conclusion, heavy smokers with the EP phenotype on QCT in Southern Brazil were more likely to have airflow limitation, worse FEV_1_, and more exacerbation-like episodes than those with the NEP phenotype. Up to nine percent of smokers with no evidence of airflow limitation on spirometry had a previous exacerbation-like episode, and 23.8% of those patients with no airway limitation were classified in the EP phenotype.

## Supporting information

S1 File. Dataset (XLSX)
